# Dietary weight loss strategies for kidney stone patients

**DOI:** 10.1007/s00345-022-04268-w

**Published:** 2023-01-02

**Authors:** Roswitha Siener, Christine Metzner

**Affiliations:** 1 Department of Urology, University Stone Center, University Hospital Bonn, Venusberg-Campus 1, 53127 Bonn, Germany; 2 Bonn Education Association for Dietetics r. A., Cologne, Germany; 3 Clinic for Gastroenterology, Metabolic Disorders and Internal Intensive Medicine (Medical Clinic III), RWTH, Aachen, Germany

**Keywords:** Urinary stones, Urolithiasis, Ketogenic diet, Energy-restricted diet with meal replacement, Mediterranean diet, DASH diet

## Abstract

**Purpose:**

Overweight has been associated with an increased risk of incident and recurrent kidney stone disease. Weight reduction is the therapeutic consequence to decrease the risk of stone formation. This review examines the effectiveness of different weight loss strategies on weight reduction and cardiometabolic risk profile, with a particular focus on risk factors for urolithiasis.

**Methods:**

A selective literature search was performed using PubMed and Cochrane library.

**Results:**

Clinical evidence for the potential benefits of dietary weight loss strategies for kidney stone disease is limited. A conventional, energy-restricted diet may significantly induce weight loss and reduce urinary supersaturation of calcium oxalate in overweight individuals with or without a history of stone formation. The current data indicate that an energy-restricted diet with partial meal replacement may additionally decrease the relative supersaturation of uric acid and further improve the cardiometabolic risk profile, and, thus, may be a favourable option for overweight kidney stone patients. Studies on the Mediterranean and DASH diets on the association between weight loss and the risk of urinary stone formation are lacking.

**Conclusion:**

An energy-restricted diet with or without meal replacement could be a promising weight loss strategy for overweight kidney stone patients. Further studies are needed to evaluate the impact of different weight loss strategies on urinary risk factors and cardiometabolic risk profile in urolithiasis.

## Introduction

Obesity, as determined by body mass index (BMI) or waist circumference (WC), is a chronic, multifactorial disease [1]. The prevalence of overweight and obesity has increased worldwide in recent decades [2]. A high BMI with excessive accumulation of body fat is associated with adverse health outcomes and comorbidities such as metabolic syndrome or its single components, cardiovascular and chronic kidney diseases [2, 3]. A systematic review with meta-analysis of cohort studies found a positive relationship between BMI and the risk of incident kidney stone disease [4]. Results of a meta-analysis also point to an association between overweight and the risk of stone recurrence [5].

Metabolic syndrome, a cluster of cardiometabolic risk factors including abdominal obesity, impaired glucose regulation, dyslipidaemia, and hypertension, is ultimately the result of a positive energy balance [1, 6]. Growing evidence suggests that metabolic syndrome is closely related to urolithiasis, and that the risk of urinary stone formation increases with the number of metabolic syndrome traits [7–11]. A bidirectional association between metabolic syndrome and non-alcoholic fatty liver disease (NAFLD) has also been established [12]. NAFLD, also referred to as the hepatic component of the metabolic syndrome, not only affects the liver but can also increase the risk of developing extra-hepatic diseases, including chronic kidney disease and urolithiasis [13, 14]. Weight loss is an essential intervention to obesity to reduce morbidity and mortality and to improve quality of life [1].

## Overweight and risk factors for urolithiasis

The propensity of patients with overweight and other metabolic syndrome traits to form stones is influenced by changes in the urinary risk profile. Overweight has been reported to significantly impact urinary risk factors for stone formation [15]. In particular, a negative association between BMI and urine pH has been repeatedly observed, both in stone-forming and non-stone-forming individuals [16–20]. A systematic review with meta-analysis revealed that overweight versus normal weight stone patients not only had lower urine pH, but also higher 24-h excretion of calcium, oxalate, uric acid, and sodium, all of which increase the risk of uric acid and calcium oxalate urolithiasis [20]. However, the exact underlying mechanisms are not yet clear. Since no association was found between BMI and urine volume, it is suggested that a higher concentration of promoters plays a crucial role in urinary stone formation in overweight patients [16, 20].

Effective weight loss strategies are required to improve cardiometabolic risk profile and reduce the risk of stone formation in kidney stone patients with overweight and other components of the metabolic syndrome. Approaches to weight reduction should be individually tailored to the cardiometabolic situation and comorbidities of the stone patient, taking into account the respective stone type. To identify underlying clinical and biochemical abnormalities, a comprehensive anthropometric, dietary and metabolic evaluation is required.

## Classification and diagnostic measures

BMI is an established, easily determined marker of overweight and obesity in adults [21]. According to the World Health Organization (WHO), a BMI ≥ 25.0 kg/m^2^ is defined as overweight, and a BMI ≥ 30.0 kg/m^2^ as obesity [1, 2]. BMI is only a score and does not reflect the body fat mass objectively, as it does not distinguish between body fat mass and other body compartments [22, 23]. In contrast, waist circumference (WC), waist-to-hip ratio (WHR), and waist-to-height ratio (WHtR) are measures of fat distribution. Visceral rather than subcutaneous fat accumulation is associated with metabolic effects [23].

An ethnic-specific European WC ≥ 80 cm in women and ≥ 94 cm in men [6] or > 88 cm in women and > 102 cm in men [24] indicates abdominal obesity. A WHR ≥ 0.85 for women and ≥ 0.90 for men is considered android fat distribution [6]. An age-independent cut-off value for WHtR in adults is given as 0.5 [25]. However, according to Schneider et al. [23], an age-dependent cut-off value for WHtR between 0.5 and 0.6 seems to better reflect cardiovascular risk. BMI, WC, WHR, and WHtR were all found to be associated with higher prevalence and incidence of kidney stone disease [26].

Indications for the medical nutrition therapy of overweight/obesity include a BMI ≥ 30 kg/m^2^ or a BMI ≥ 25 kg/m^2^ and the presence of obesity-related comorbidities [27]. In individuals with a BMI between 25.0 and 29.9 kg/m^2^ without overt comorbidities, the goal should be to prevent further weight gain through dietary advice and physical activity [27]. Weight loss objectives should be realistic, individualized and aimed at long-term, also taking into account age progression. A detailed and standardized medical history should always be obtained before starting obesity therapy, including recording of concomitant diseases and reasons for failed diet attempts. The dietary behaviour of the patient can provide important clues to the causes of overweight. Self-reported methods based on recall, such as food frequency questionnaires or 24-h dietary recalls, or based on real-time recordings, e.g. food diaries, in combination with objective nutritional biomarkers, are useful options for assessing the dietary habits of kidney stone patients [28]. In addition to the type and quantity of food consumed, the timing of meals and the type and volume of beverages consumed should also be recorded.

## Bariatric surgery—therapy or risk?

The dominant bariatric surgical procedures are sleeve gastrectomy and Roux-en-Y gastric bypass, accounting for approximately 90% of all operations worldwide [29]. Bariatric surgery is considered an effective treatment option for severe obesity, resulting in efficient and sustained weight loss and a reduction in obesity-associated comorbidities such as type 2 diabetes, and hypertension [29–31]. Bariatric surgery may also be associated with a lower risk of kidney function decline and may prevent and slow the progression of chronic kidney disease in severely obese patients [32]. Findings suggesting an improvement of metabolic disease led to the concept of metabolic surgery and recommendations to lower thresholds for considering surgery in patients with metabolic disorders [29, 31]. Metabolic and bariatric surgery is currently recommended for individuals with a BMI ≥ 35 kg/m^2^, regardless of the presence, absence, or severity of comorbidities and for individuals with BMI of 30.0–34.9 kg/m^2^ and metabolic disease, especially type 2 diabetes [29].

However, bariatric surgical procedures such as Roux-en-Y gastric bypass, one of the most common surgical procedures in recent years, one anastomosis gastric bypass, and biliopancreatic diversion/duodenal switch are associated with an increased risk of stone formation in patients with or without a previous history of urolithiasis [33–35], which has been mainly attributed to increased urinary oxalate excretion [34–37]. Even after sleeve gastrectomy, a significant increase in urinary supersaturation of calcium oxalate was observed [38]. Lifelong replacement therapy and monitoring of protein, vitamin and mineral deficiencies are required, particularly after malabsorptive surgery, and management of dumping syndrome, gastro-oesophageal reflux, and hypoglycaemia can be challenging to treat [31]. Dietary strategies are therefore more appropriate for weight loss, also in patients at risk for kidney stone formation.

## Overweight management

The primary goal of weight management is to reduce health risks, especially cardiovascular and cardiometabolic risks [27]. Lifestyle changes represent a cornerstone in obesity management, and prevent diabetes mellitus and its cardiovascular complications [39]. The intervention programs are based on three pillars, namely diet, physical activity and behavioural therapy. The latter leads in particular to a weight loss of 5–15% of the initial body weight, which is associated with a reduction in cardiovascular disease and an improvement in quality of life [40]. A prerequisite for weight reduction is a negative energy balance due to a lower energy intake and/or a higher energy expenditure, while ensuring adequate nutrient supply. To achieve a significant weight reduction, the energy deficit must be at least 500 kcal/day below energy requirements or a dietary plan of 1500–1800 kcal/day for men and 1200–1500 kcal/day for women must be followed [31]. The goal of dietary therapy for overweight, in addition to weight loss, is to achieve a long-term change in dietary habits. When choosing a diet, attention should be paid to concomitant diseases and underlying metabolic disorders.

## Very low-energy diets

Very low-energy diets (VLED), providing fewer than 800 kcal/day, are used to induce rapid weight loss [41]. In patients with morbid obesity (BMI > 40 kg/m^2^) requiring severe weight loss while maintaining lean body mass, specially formulated VLED meal replacements may be used in place of all meals [42]. The composition of these formula diets is regulated according to the food authorities of the respective countries, e.g. the European Food Safety Authority (EFSA) and the U.S. Food and Drug Administration (FDA), and/or expert associations. Alternatively, protein may be obtained from a modified fast, consisting of servings of lean fish, meat, and poultry, and must be supplemented with a multivitamin and 2–3 g/day potassium [41]. Since side effects are to be expected without sufficient supplementation, VLEDs with meal replacement should be preferred as they are more likely to ensure adequate supply. Studies have shown that formula VLEDs and low-energy diets (LEDs) can achieve clinically significant weight loss of between 10 and 15% body weight for up to 12 months, and when combined with weight loss maintenance strategies can aid long-term weight maintenance for up to 4 years [43]. To our knowledge, studies on the effects of VLEDs on weight loss, urinary and cardiometabolic risk factors in overweight kidney stone patients are lacking.

## Very-low-carbohydrate or ketogenic diets

Ketogenic diets include high fat, moderate or high protein, and very low carbohydrate intake. Ketogenic diets are popular for weight loss, but are also used as a therapy for epilepsy in children [44]. The Atkins diet may be the most popular ketogenic diet. It is anticipated that under hypoglycaemic conditions of a ketogenic diet, acetyl-CoA produced from the β-oxidation of fatty acids is converted into ketone bodies, resulting in a metabolic acidosis [45]. Acidosis is likely to promote not only a decrease in urinary pH and citrate excretion, but also an increase in urinary calcium excretion, aggravating the risk of uric acid and calcium oxalate stone formation [46, 47]. A study by Reddy et al. [48] showed similar alterations in urinary pH, citrate and calcium excretion in healthy subjects on a low-carbohydrate protein-rich diet. Interestingly, urinary uric acid excretion did not change under these diets [46, 48]. A systematic review with meta-analysis revealed an estimated incidence of kidney stones of 5.8% in children and 7.9% in adults on ketogenic diets [44]. Due to the increased risk for urinary stone formation [46–48], ketogenic diets appear to be unsuitable as a weight loss strategy for overweight kidney stone patients. To prevent kidney stone formation in patients on a ketogenic diet, adequate fluid intake and urine alkalinization using oral alkali citrate should be considered [46–48].

## Low-carbohydrate and low-fat diets

There is no internationally accepted definition for low-carbohydrate diets (LCD) and low-fat diets (LFD). The hallmark of an LCD is a carbohydrate intake of 40–50% and a fat intake of 40–45% of total energy, whereby the carbohydrate and fat quality is decisive. In contrast, an LFD desirably reduces total intake of fat, especially saturated and trans- fatty acids. At the same time, however, less unsaturated fatty acids such as monounsaturated and omega-3 fatty acids are consumed, which has a negative effect. A randomized trial comparing four diets with different proportions of carbohydrates and fat found modest statistically significant weight loss after one year on each diet, with no statistically significant differences between diets [49]. Although LCDs and LFDs result in similar weight loss, an LCD is more suitable, because an LFD typically reduces or fails to increase HDL cholesterol levels. Studies on the effects of LCDs and LFDs on the risk of urinary stone formation are lacking.

## Intermittent fasting

Intermittent fasting (IF) refers to regular periods of no or very limited energy intake, usually consisting of a daily fast of 16 h, a 24-h fast on alternate days, or a fast two days per week on non-consecutive days. A systematic review found that IF may be a promising strategy for the treatment of overweight and obesity, although little is known about long-term sustainability and health effects [50]. However, it is still unclear whether IF provides additional cardiometabolic benefits as compared to continuous daily energy restriction [51]. The effects of IF on weight loss, cardiometabolic and urinary risk factors of overweight kidney stone patients have not yet been studied.

## Energy-restricted, modified diet

Another weight loss strategy is a conventional, lacto-vegetarian-oriented mixed diet with a moderate energy deficit. Within this diet, red and processed meats, saturated fat, trans-fatty acids, and salt intake should be limited, while white meat, fat-reduced dairy products and vegetable oils should be prioritized. Fruit, non-starchy vegetables, legumes, wholegrain products, and nuts should be preferred [52]. A randomized trial revealed that an energy-restricted diet reduced body weight and several cardiometabolic risk factors at one year to a similar extent as any of the other three diets studied [49]. Increased adherence was associated with greater weight loss and cardiometabolic risk factor reductions for each diet group.

Unfortunately, clinical evidence for the benefits of an energy-restricted, modified diet on weight loss, urinary and cardiometabolic risk profile in overweight kidney stone patients is limited. A study of 39 adult patients with idiopathic calcium oxalate stone disease and a BMI ≥ 30 kg/m^2^ evaluated the effects of an energy-restricted diet on weight loss, cardiometabolic and urinary risk factors [53]. The median energy intake was 1300 kcal/day with a macronutrient distribution of 55% carbohydrates, 15% protein and 30% fat of the total energy intake. No changes in 24-h urinary parameters were observed after a 12-week energy-restricted diet, except for a significant increase in urine volume and a decrease in urinary calcium oxalate supersaturation. Apart from modest weight loss, no changes in cardiometabolic risk factors were observed.

## Energy-restricted modified diet with meal replacement

The partial meal replacement (MR) strategy is usually to replace one or two main meals per day of an energy-restricted diet with low-energy formula products, which are commercially available products fortified with vitamins and minerals, including liquid formula, powdered mixes, energy bars and prepackaged foods. A systematic review and meta-analysis of randomized controlled trials revealed that the effect of MR-based energy-restricted diet on weight loss was superior to the effect of food-based energy-restricted diet [54]. In addition, the findings suggested that ≥ 60% of total daily energy intake from MR had the greatest effect on weight loss.

A randomized, controlled study evaluated the effect of a conventional energy-restricted, lacto-vegetarian-oriented mixed diet with or without MR on cardiometabolic risk profile and urinary risk factors for kidney stone formation in overweight women without a history of urolithiasis [55]. Although both dietary interventions resulted in a significant weight reduction, relative weight loss and rate of responders were higher in the MR group. Weight loss improved cardiometabolic risk profile in both groups. While the relative supersaturation of calcium oxalate decreased significantly in both groups, a significant decline in serum uric acid concentration and relative supersaturation of uric acid was observed only in the MR group. Finally, the energy-restricted modified diet with MR showed significant advantages over the energy-restricted modified diet alone. Further studies are needed to evaluate the impact of an energy-restricted diet with MR on urinary risk factors and the cardiometabolic risk profile of overweight kidney stone patients.

## Mediterranean diet

The Mediterranean diet is characterized by a high consumption of vegetables, legumes, fruits, wholegrain products, fat from olive oil and nuts, and moderate intake of fish, and poultry, and a low intake of dairy products and red meat. A systematic review of randomized controlled trials on the long-term effects of the Mediterranean diet on weight loss compared to a low-fat diet, a low-carbohydrate diet, and the American Diabetes Association diet found that the Mediterranean diet resulted in greater weight loss than the low-fat diet at ≥ 12 months, but similar weight loss as other comparator diets in overweight or obese individuals trying to lose weight [56]. Moreover, the Mediterranean diet was generally similar to comparator diets at improving other cardiovascular risk factors, including blood pressure and lipid levels. In the PREDIMED (Prevención con Dieta Mediterránea) study involving subjects at high cardiovascular risk, the incidence of major cardiovascular events was lower among those assigned to an energy-unrestricted Mediterranean diet supplemented with extra-virgin olive oil or nuts than among those assigned to a reduced-fat diet [57].

Studies on the effect of the Mediterranean diet on the risk of stone formation showed mixed results. While adherence to a Mediterranean dietary pattern was associated with a lower risk of incident kidney stone formation in two cohort studies [58, 59], another study of overweight individuals failed to find an association between adherence to the Mediterranean dietary pattern and risk for urolithiasis [60]. Although higher consumption of plant foods may lead to increased dietary oxalate intake and urinary excretion of oxalate, a major risk factor for calcium oxalate stone formation, higher intake of fruits and vegetables may also increase urinary pH and citrate excretion, which may partly offset the elevated urinary oxalate excretion [61, 62]. In particular, attention should be paid to an adequate calcium intake to reduce intestinal oxalate absorption. Further studies are needed to investigate the effects of a Mediterranean dietary pattern on weight loss, urinary risk factors and cardiometabolic risk profile in overweight stone formers.

## Dietary approaches to stop hypertension diet

The Dietary Approaches to Stop Hypertension (DASH) diet is rich in vegetables, fruits, legumes, nuts and wholegrain products, moderate in low-fat dairy products, and limited in sugar, red and processed meats, fat, and sodium chloride. The most relevant difference between the Mediterranean and the DASH diet is the emphasis of the former on unsaturated fatty acids, mainly from extra-virgin olive oil [52]. An umbrella review of systematic reviews and meta-analyses of the relation of the DASH dietary pattern with cardiometabolic outcomes in individuals with and without diabetes concluded that the DASH dietary pattern is associated with decreased incidence of cardiovascular disease and improves blood pressure with evidence of other cardiometabolic advantages [63].

A prospective study in three large cohorts indicated that consumption of a DASH-style diet is associated with a marked decrease in the risk of incident kidney stones [64]. A randomized controlled trial of 57 recurrent stone formers with hyperoxaluria revealed a significant increase in urinary pH, citrate and magnesium excretion, but no significant change in urinary oxalate excretion and calcium oxalate supersaturation with a DASH-style diet versus a low-oxalate diet [65]. A possible explanation for the opposite trends in urinary oxalate excretion versus calcium oxalate supersaturation on the DASH diet compared to the low-oxalate diet is the high calcium intake on the DASH diet, resulting in more calcium available in the intestinal lumen to form a complex with oxalate, thus reducing its absorption. Further research is needed to evaluate the effect of a DASH diet on weight loss, cardiometabolic and urinary risk factors in overweight kidney stone formers.

## Physical activity

A fundamental cause of overweight and obesity is an increase in physical inactivity due to increasingly sedentary nature of many forms of work, increasing urbanization, and changing mode of transportation [2]. Regular physical activity has a beneficial effect on the energy balance, although it is not firmly established that exercise contributes significantly to weight loss. Despite the modest effect of physical activity on weight loss, physical activity should be recommended as part of a healthy lifestyle. Encouraging individuals to exercise for longer periods of time each day may help to enhance weight loss [66]. In addition to an increase in energy expenditure due to the additional muscle work, there is a higher basal metabolic rate in the long term as a result of an increase in muscle mass. Especially in low-energy diets, the loss of muscle mass can be reduced by regular physical activity, so that the decrease in basal metabolic rate is less pronounced.

## Conclusion

Obesity and other metabolic syndrome traits were found to increase the risk of urinary stone formation. Effective dietary weight loss strategies are required to reduce the risk of urinary stone formation and to improve the cardiometabolic risk profile in overweight kidney stone patients. To identify underlying abnormalities, a comprehensive anthropometric, dietary and metabolic evaluation is required. Clinical evidence for the potential benefits of dietary weight loss strategies for urolithiasis is limited. With a conventional, energy-restricted diet, a significant weight loss and reduction in the urinary supersaturation of calcium oxalate can be achieved in overweight individuals with and without a history of stone formation. An energy-restricted diet with partial MR may additionally contribute to a significant decrease in serum uric acid concentration and relative supersaturation of uric acid. Moreover, an energy-restricted diet with MR may further improve the cardiometabolic risk profile, and, thus, may be a favourable option for overweight kidney stone patients. Further research is needed to examine the effects of different dietary weight loss strategies on urinary risk factors and cardiometabolic risk profile in overweight kidney stone patients.

## Data Availability

Data are available upon reasonable personal request.
